# 1182. Impact of Penicillin Allergy Focused Antimicrobial Stewardship Interventions on Antimicrobial Use and Allergy Documentation - Optimizing Allergy Prescribing Alerts and Pharmacy Allergy Assessments

**DOI:** 10.1093/ofid/ofad500.1022

**Published:** 2023-11-27

**Authors:** Emily Stephens, Jennifer Riney, Yvonne J Burnett

**Affiliations:** Missouri Baptist Medical Center, St. Louis, Missouri; Missouri Baptist Medical Center, St. Louis, Missouri; University of Health Sciences and Pharmacy in St. Louis/Missouri Baptist Medical Center, St. Louis, Missouri

## Abstract

**Background:**

The discrepancy between true prevalence and reported penicillin (PCN) allergies results in suboptimal antimicrobial use, which is associated with increased adverse drug reactions, antimicrobial resistance, increased hospital length of stay, and poor outcomes. Many patients with mild-moderate reactions tolerate other beta-lactams, but allergy records are often inaccurate or missing information. In 2021, our institution began suppressing prescriber-facing PCN allergy alerts when ordering cephalosporins for patients with mild-moderate PCN reactions. More recently, a pharmacist-driven allergy assessment via chart review +/- patient interview was piloted to clarify PCN allergy documentation. This quasi-experimental study evaluated the effect of PCN allergy interventions on antimicrobial use.

**Methods:**

Days of broad-spectrum antimicrobials in patients with PCN allergies was compared between groups [pre-intervention (G1, n=100), post-alert suppression (G2, n=100), and allergy assessment (G3, n=50)]. Patients were excluded for cephalosporin allergy, MRSA or ESBL history, surgical prophylaxis or UTI indication, or antibiotics within 48 hours of mechanical ventilation. Groups were matched 2:2:1 based on length of stay and antibiotic indication. Additional outcomes included cephalosporin use, allergy clarifications, and time spent per allergy assessment.

**Results:**

The most common antibiotic indications were pneumonia (54%) and intra-abdominal infection (28%). No differences were noted in median duration of broad-spectrum therapy (2 vs 1 vs 1 days, p=0.52) or cephalosporin therapy (1 vs 2 vs 3 days, p=0.39). However, more patients received cephalosporins as initial therapy post-alert suppression, G1 40 (40%) vs G2 57 (57%), p=0.02 vs G3 26 (52%). A pharmacist completed 50 allergy assessments (50 chart review with 19 interviews) and clarified 43 (86%) records to include previous beta-lactam tolerance. Average time of chart review and interview was 4 minutes each.

**Conclusion:**

PCN allergy alert suppression significantly increased initial cephalosporin use and decreased broad-spectrum use. Pharmacist allergy assessment improved allergy documentation accuracy, but future study is required to assess the clinical impact on antimicrobial use.

**Disclosures:**

**All Authors**: No reported disclosures

